# Association between mental health self-care status and well-being-promoting behaviors among university students living in rural areas

**DOI:** 10.1371/journal.pone.0334221

**Published:** 2025-10-15

**Authors:** Airi Endo, Sayuri Goryoda

**Affiliations:** Laboratory of Integrated Food and Agricultural Science, Department of Food, Life and Environmental Sciences, Yamagata University Faculty of Agriculture, Tsuruoka-shi, Yamagata, Japan; Alexandria University Faculty of Nursing, EGYPT

## Abstract

This study examined the relationship between the stages of behavioral changes related to mental health self-care and well-being (WB)-promoting behaviors. Students residing in a local city were invited to participate in an anonymous survey administered using Google Forms between Saturday, July 29, and Friday, August 4, 2023. The “Well-Being Promoting Behavior Inventory” was used to measure WB-promoting behaviors, and the respondents were asked to mark their responses. The scores for each of the four items were added (range, 32–160), and a Poisson regression analysis was conducted. The sum of these Well-Being promoting behavior scores was used as the dependent variable. The scores are non-negative integers and have the characteristics of count data. Since the variance was approximately equal to the mean, a Poisson regression model was deemed appropriate. The model’s assumptions, such as equivariance, were also evaluated and found to be reasonably satisfied. The stage of behavioral changes related to mental health self-care served as the explanatory variable, and the presence of worries or stress and sex were the adjustment variables. The mean age of the 113 subjects analyzed was 20.7 ± 1.42 years, with 51 men and 62 women. The distribution of behavioral change stages was 17.7%, 54.9%, 13.3%, 2.7%, and 11.5% in precontemplation, contemplation, preparation, action, and maintenance stages, respectively. Even after adjusting for the presence or absence of stress and sex, the proportion of those with more frequent WB-promoting behaviors was significantly higher in the “ Contemplation, Preparation, Action, and Maintenance” phase than in the “Precontemplation” phase (prevalence ratio [PR]=1.07, 95% confidence interval [CI]=1.02–1.13). Our results suggest an association between the behavior change stage in mental health self-care and WB-promoting behaviors. It may also be helpful to assist individuals in transitioning from the precontemplation stage to the contemplation stage when promoting behavioral changes related to mental health self-care, as significant differences were found after the contemplation stage. In the future, we believe that a larger sample size will be necessary for similar follow-up studies, to improve reliability and conduct a stratified analysis in a more detailed study.

## Introduction

In recent years, there has been a growing interest in the concept of well-being (WB). According to the Ministry of Health, Labor and Welfare, WB is “a concept that refers to a person’s physical, mental, and social well-being, in which individual rights and self-actualization are guaranteed”. Within the concept of well-being [1], people with low “Psychological Well-being (PWB) [2],” a positive psychological function, have a higher risk of developing depression in the future [3]. For instance, patients suffering from schizophrenia, typically self-report lower PWB levels than healthy individuals [4]. The number of outpatients with mental illnesses is rising in Japan [5], and mental illness is no longer uncommon, even among college-age students [6]. Early detection and preventive interventions are crucial for university students because mental health problems can affect not only their adjustment to student life but also their future lives after leaving university [7]. In this context, “self-care”, which represents general actions that one can practice as mental health measures, is significant. However, measures specific to individual contexts are even more crucial. “Self-care” can also be defined as any activities that individuals initiate and continue to maintain and improve their health and WB [8]. There is no clear consensus regarding the nature of proactive preventive mental health-related measures that can be taken by individuals to improve their mental health, even though there have been advancements in coping strategies for individuals with mental health problems, including elements such as institutional and environmental improvements [9]. The “mental health promotion and universal prevention strategies” released by the World Health Organization (WHO) include building individual resources for mental health—including the ability to practice self-care and make behavioral and lifestyle choices to maintain good mental health in the context of “ability to live, learn, and work effectively” [10]. Therefore, we believe that self-care is a crucial tool to maintain and improve mental health, thus preventing mental health issues.

The WB-Promoting Behavior Inventory summarizes specific behaviors that are generally perceived as promoting PWB [11]. Using this database, one short-term cognitive-behavioral program to increase PWB showed that, while PWB was higher after the program’s implementation and remained high one month later, the methods used to promote behavioral changes were not properly implemented [12]. Interventions increase effectiveness via a stage-based approach [13]. In order to effectively promote behavioral changes, it is necessary to identify an individual’s current stage and tailor interventions accordingly [14]. However, the relationship between mental health self-care status and behaviors that promote PWB among college students remains unclear. It has been suggested that high PWB may lead to the prevention of mental health issues [2,3]. Therefore, we believe that the implementation of “WB-promoting behaviors”, which summarize the behaviors in which PWB is strongly felt, can be included in the category of mental health self-care activities.

In this context, this study aimed to examine the relationship between stages of behavioral changes and WB-promoting behaviors related to mental health self-care among Japanese university students.

## Materials and methods

### Survey subjects

Our survey was administered to undergraduate and graduate students residing in a local city, using Google Forms (Google LLC, Mountain View, CA, USA), as an anonymous questionnaire. The survey period was from July 29 to August 4, 2023.

### Survey contents

#### Basic attributes.

Sex, age, and the presence or absence of worries and stress were surveyed. The presence or absence of worries and stress was determined using question 9: “Do you currently have worries or stress in your daily life?” which was taken from the “National Survey of Living Conditions (Health Questionnaire),” 2022 [15].

#### Well-being Promoting Behavior Inventory.

This scale was created by Iwano et al. to measure the frequency of “behaviors that confer a sense of psychological well-being” [11]. It consists of four subscales, 32 items in total: “interpersonal support behaviors,” “task performance behaviors,” “self-determined behaviors,” and “challenging behaviors.” Total scores can be calculated and analyzed for each subscale, as well as for all four subscales.

#### Behavior change stage in mental health self-care.

The behavior change stage in mental health self-care was evaluated based on Prochaska et al.’s Transtheoretical Model (TTM) [16], the evaluation was conducted. The TTM is a psychological framework that comprehensively captures the process of individual behavior change and consists of several theoretical constructs. One of its core components is the “Stages of Change.” These stages conceptualize behavior change not as a singular event, but as a dynamic process involving progression through multiple phases. Respondents were categorized into one of the five stages of behavior change, defined as follows: “*Not interested*” (precontemplation), “*Interested, but not planning to take action immediately* ” (contemplation), “*Interested and about to start taking action*” (preparation), “*Have changed behavior within the past six months*” (action), and “*Have maintained the behavior change for more than six months*” (maintenance).

#### Statistical Analysis.

Poisson regression analysis was conducted with the total score of frequency of Well-Being Promoting Behavior as the objective variable, the stages of behavior change in mental health self-care as the explanatory variable, and the presence of stress and sex as adjustment variables. Poisson regression analysis is an appropriate statistical technique when the distribution of the objective variable does not assume normality and the count data take only positive values. IBM SPSS ver. 29 (IBM Corporation, Armonk, NY, USA) was used for the analysis, and the significance level was set at p = 0.05 on two-tailed Student’s t-tests.

#### Ethical considerations.

Every response was obtained from a consenting party after the latter was asked to review a disclaimer stating that a response was not mandatory and that no personal information would be collected. This study was approved by the Yamagata University School of Medicine Ethics Review Committee (2022−335). Written informed consent was obtained from the study participants.

## Results

The characteristics of the participants are shown in Table 1. Of the 117 participants who responded to the questionnaire, 113 were included in the analysis, excluding those with missing values and other data. Their mean age was 20.7 ± 1.42 years, and the sex distribution was 51 men and 62 women. Of these, 69% indicated that they had worries or stress, while 31% did not. The distribution of their behavioral change stages was 17.7% in the precontemplation stage, 54.9% in the contemplation stage, 13.3% in the preparation stage, 2.7% in the action stage, and 11.5% in the maintenance stage.

**Table 1:** Characteristics of participants (n = 113).

		%
Gender	Men	45.1
	Women	54.9
Age		20.7 ± 1.4
Worries · Stress	No	31.0
	Yes	69.0
Well-being Promoting Behavior Inventory	<average	46.0
	≥average	54.0
Supporting Behavior toward Others	<average	37.2
	≥average	62.8
Task-Completing Behavior	<average	33.6
	≥average	66.4
Self-Determination Behavior	<average	45.1
	≥average	54.9
Challenging Behavior	<average	48.7
	≥average	51.3
Behavior Change Stages of Self-Care For Mental Health	precontemplation	17.7
	contemplation	54.9
	preparation	13.3
	action	2.7
	maintenance	11.5

Table 2 shows the results of the Poisson regression analysis of mental health self-care behavior change stages and WB-promoting behaviors. Even after adjusting for sex and stress status, with respect to the frequency of WB-promoting behaviors, the proportion of those who belonged to the contemplation, preparation, action, and maintenance stage who had a higher frequency of WB-promoting behaviors was significantly higher than those who belonged to the precontemplation stage (prevalence ratio [PR]=1.07, 95% confidence interval [CI]=1.02–1.13).

**Table 2:** Prevalence ratios for well-being promoting behavior and related factors using Poisson regression analysis.

	PR	95%CI
Behavior change stages of Self-care for mental health		
Contemplation · Preparation · Action · Maintenance	1.07	1.02–1.13
Precontemplation	1.00	(reference)
Worries · Stress		
Yes	1.06	1.06 - 1.10
No	1.00	(reference)
Gender		
Men	1.03	0.99 - 1.07
Women	1.00	(reference)

CI, confidence interval; PR, prevalence ratio.

PR were adjusted for Worries · Stress and Gender.

The results of our Poisson regression analysis regarding behavior change stages and challenging behaviors in mental health self-care are presented in Table 3. Among the four items describing WB-promoting behaviors, significant differences were found only in terms of challenging behaviors that differed between the contemplation, preparation, action, and maintenance stage group vs the precontemplation one (PR = 1.25, 95% CI = 1.12–1.39).

**Table 3:** Prevalence ratios for challenging behavior and related factors using Poisson regression analysis.

	PR	95%CI
Behavior change stages of Self-care for mental health		
Contemplation · Preparation · Action · Maintenance	1.25	1.12 - 1.39
Precontemplation	1.00	(reference)
Worries · Stress		
Yes	1.07	1.00 - 1.19
No	1.00	(reference)
Gender		
Men	1.07	0.99 - 1.15
Women	1.00	(reference)

CI, confidence interval; PR, prevalence ratio.

PR were adjusted for Worries · Stress and Gender.

The analysis with the addition of interaction terms for gender and presence of stress also confirmed significant differences in the stage of behavior change and challenging behavior (PR = 1.37, 95% CI = 1.09–1.73). This suggests that the individual’s interest in mental health self-care is an important factor in increasing the frequency of challenging behaviors.

## Discussion

This study aimed to examine the association between the stages of behavioral change regarding mental health self-care and WB-promoting behaviors among university students. Our results suggest a significant association between the stage of behavioral change in mental health self-care and WB-promoting behaviors. Notably, significant differences were observed across the stages of behavioral change in mental health self-care with respect to challenging behaviors. Furthermore, when the interaction terms of gender and the presence or absence of stress were included in the analysis, significant differences in challenging behaviors were again confirmed. These findings suggest that an individual’s interest in mental health self-care is a key factor in increasing the frequency of challenging behaviors. Previous studies have stated that it is necessary to promote the advancement of college students who are in the precontemplation or contemplation stage [17], or lower stages (i.e., “not feeling the need for behavioral changes” and “hesitation and procrastination toward behavioral changes”) to advance to higher stages of the process, to achieve and establish healthy lifestyles [18]. Therefore, those in the contemplation, preparation, action, and maintenance stage are regarded as actively acquiring knowledge regarding mental health self-care methods and initiating and implementing actions. Conversely, those in the precontemplation and contemplation stages are considered not to feel the need for mental health self-care, feel burdened by it, or do not know what to do. Therefore, the percentage of students with a high frequency of WB-promoting behaviors was significantly higher in the contemplation, preparation, action, and maintenance stage than in the precontemplation one. It may also be significant to consider how to transition from the interest phase to the readiness phase when promoting behavioral changes regarding mental health self-care. Related to mental health measures in college students, in an examination of self-help strategies focused on adolescents, Takenaka (2019) found that the content was simple, the practices were related to the lifestyles of their adolescent cohort, and they could be integrated into daily life without the need for any excessive effort [19]. Nagashima (2021) stated that self-help strategies are actions that can be taken to help prevent mental health issues and represent a means of self-care that focuses on coping to maintain and promote mental health [20]. From this perspective, interpersonal helping behaviors, task performance behaviors, and self-determination behaviors can be incorporated into or experienced by university students in their daily lives, while challenging behaviors can be viewed as requiring particular efforts. Regarding interpersonal helping behaviors, prosocial behaviors such as helping behaviors are considered relevant. Prosocial behavior is defined as behavior that benefits others [21] and has been observed in all cultures [22]. This may explain why there was no significant difference between those in the contemplation, preparation, action, and maintenance group vs those in the precontemplation group in our research. The task-oriented behaviors in our study consisted of items such as “I tried to complete reports and assignments” and “I worked seriously on work and schoolwork.” For our research of university students, task-oriented behaviors overlapped with other behaviors they engaged in during their daily lives—such as studying for tests and research activities [11]. Thus, there were no significant differences between the stages of behavioral change. Therefore, we determined that there were no significant differences in terms of behavioral change stage. We believe that self-determined behavior can be viewed from the perspective of independence, which is an important developmental issue in adolescence. Yamada (2011) considered independence from the perspective of psychological development in adolescence and defined it as “to manage and decide one’s own feelings, thoughts, and actions independently, and to take responsibility for them” [23]. In the context of this study, the term “self-determined behavior” was defined as “the behavior of deciding things based on one’s own value standards and respecting one’s own will.” It included items such as “deciding things based on one’s own ideas, without being influenced by the opinions of others” and “making a decision by one’s self when faced with a choice.” Since self-determined behavior was related to independence -an important developmental issue in adolescence [11] there was no significant difference between those who were in the contemplation, preparation, action, and maintenance group vs those who were in the precontemplation one. The fact that we were able to clarify the distributions of behavioral change stages in the context of self-care for mental health among undergraduate and graduate students living in regional cities, may contribute to directing future efforts concerning mental health in university settings.

Although this study is significant, it was also subject to several key limitations worth noting. The first is its sample size. Because only 113 participants responded to our survey, future related studies should strive to analyze larger sample sizes to increase the reliability of their results. In addition, the sample size was small when divided by behavioral change stage, so stratified analyses by stage were not possible. However, the analysis with the addition of an interaction term indicates that the individual’s interest in mental health self-care is significantly related to the frequency of challenging behavior, even after adjusting for general psychological background variables such as sex and stress.

It may indicate a certain generalizable psychological mechanism, and the generalizability is relatively high in similar environments and cultures. On the other hand, there may be limitations in generalizing the results to people from different social backgrounds.

Second, there is a lack of uniformity in the categorization of behavioral change stages. Suwa and Sakai (2019) stated that it would be easier to obtain the effects of necessary support if the stages were divided by awareness and behavior [24]. Specifically, the current stages are described as follows: a period when there is no interest in behavioral change (precontemplation stage); a period when there is interest in behavioral change but no intention to implement it yet (contemplation stage); a period when there is a desire to implement actions for behavioral change (preparation stage); a period when clear behavioral change is observed; and a period when clear behavioral change is observed but there is no confidence in sustaining it (action stage), and a period when a clear behavioral change is observed, and there is confidence in sustaining it (maintenance stage) [24]. On the other hand, previous evaluations of behavioral change stages have often classified them according to the timing of implementation and the passage of time. Since these variables were combined in this study, we recommend that future related studies unify the evaluation of behavioral change stages into either timing and passage of time of implementation or state of awareness and behavior. Alternatively, both could be used as survey items to investigate the relationship between behavioral change stages and WB-promoting behaviors in an integrated manner.

Third, because this was a cross-sectional study, we cannot determine the causal relationship between behavioral change stages and WB-promoting behaviors related to mental health self-care. In the future, we believe that longitudinal studies should be conducted to clarify the relationship between behavioral change stages, WB-promoting behaviors, and PWB.

Fourth, this study did not assess the mental health status of the participants; therefore, we were unable to identify an association between mental health status, behavioral change stages, and WB-promoting behaviors related to mental health self-care. We believe that addressing these limitations in future research could improve understanding of mental health self-care and help identify those who would benefit most from self-care, thereby informing effective interventions.

## Conclusion

Our results suggest an association between the behavior change stage in mental health self-care and WB-promoting behaviors. It may also be helpful to assist individuals in transitioning from the precontemplation stage to the contemplation stage when promoting behavioral changes related to mental health self-care, as significant differences were found after the contemplation stage. In the future, we believe that a larger sample size will be necessary for similar follow-up studies to improve reliability and conduct a stratified analysis in a more detailed study.
